# Molecular and Clinical Links between Drug-Induced Cholestasis and Familial Intrahepatic Cholestasis

**DOI:** 10.3390/ijms24065823

**Published:** 2023-03-18

**Authors:** Giovanni Vitale, Alessandro Mattiaccio, Amalia Conti, Sonia Berardi, Vittoria Vero, Laura Turco, Marco Seri, Maria Cristina Morelli

**Affiliations:** 1 Internal Medicine Unit for the Treatment of Severe Organ Failure, IRCCS Azienda Ospedaliero-Universitaria di Bologna, 40138 Bologna, Italy; giovanni.vitale@aosp.bo.it (G.V.); sonia.berardi@aosp.bo.it (S.B.); vittoria.vero@aosp.bo.it (V.V.); mariacristina.morelli@aosp.bo.it (M.C.M.); 2 European Reference Network on Hepatological Diseases (ERN RARE-LIVER), 20246 Hamburg, Germany; 3 U.O. Genetica Medica, IRCCS Azienda Ospedaliero-Universitaria di Bologna, 40138 Bologna, Italy; alessandro.mattiaccio@unibo.it (A.M.); amalia.conti@aosp.bo.it (A.C.); marco.seri@unibo.it (M.S.); 4 Department of Medical and Surgical Sciences (DIMEC), Alma Mater Studiorum—University of Bologna, 40126 Bologna, Italy

**Keywords:** drug-induced cholestasis, idiosyncratic drug-induced liver injury, MDR1 protein, MRP2, BSEP protein, MDR3 protein, *ABCB1*, *ABCC2*, *ABCB11*, *ABCB4*, familial intrahepatic cholestasis

## Abstract

Idiosyncratic Drug-Induced Liver Injury (iDILI) represents an actual health challenge, accounting for more than 40% of hepatitis cases in adults over 50 years and more than 50% of acute fulminant hepatic failure cases. In addition, approximately 30% of iDILI are cholestatic (drug-induced cholestasis (DIC)). The liver’s metabolism and clearance of lipophilic drugs depend on their emission into the bile. Therefore, many medications cause cholestasis through their interaction with hepatic transporters. The main canalicular efflux transport proteins include: 1. the bile salt export pump (BSEP) protein (*ABCB11*); 2. the multidrug resistance protein-2 (MRP2, *ABCC2*) regulating the bile salts’ independent flow by excretion of glutathione; 3. the multidrug resistance-1 protein (MDR1, *ABCB1*) that transports organic cations; 4. the multidrug resistance-3 protein (MDR3, *ABCB4*). Two of the most known proteins involved in bile acids’ (BAs) metabolism and transport are BSEP and MDR3. BSEP inhibition by drugs leads to reduced BAs’ secretion and their retention within hepatocytes, exiting in cholestasis, while mutations in the *ABCB4* gene expose the biliary epithelium to the injurious detergent actions of BAs, thus increasing susceptibility to DIC. Herein, we review the leading molecular pathways behind the DIC, the links with the other clinical forms of familial intrahepatic cholestasis, and, finally, the main cholestasis-inducing drugs.

## 1. Introduction

Idiosyncratic Drug-Induced Liver Injury (iDILI) represents an ever-present topic among liver diseases since the development of new drugs with different mechanisms of action in every field of medicine is constantly expanding and reports of liver toxicity are becoming more and more frequent.

Indeed, iDILI accounts for more than 40% of hepatitis in adults over 50 years and more than 50% of acute liver failure (ALF). The costs of DILI for national health systems are expensive. For example, studies conducted on patients with ALF have an iDILI etiology in 11% of cases (excluding acetaminophen overdose), and 64% die or undergo liver transplantation (LT) [1]. iDILI is the most frequent indication of LT in Europe and the US and the first cause of ALF [2].

The incidence and relevance of iDILI are continuously increasing due to the ongoing development of new drugs, the increase in life expectancy, the prescription of polytherapy in the elderly, and the use of herbal products (which cause the so-called Herb-Induced Liver Injury, HILI) [2].

The diagnosis of iDILI is mainly based on the exclusion of other causes of hepatitis, on a detailed medical history, on the temporal correlation with potentially hepatoxic drugs, and on the use of algorithms and probability scores such as the Roussel Uclaf Causality Assessment Method (RUCAM) [3,4]. 

**Figure 1:**
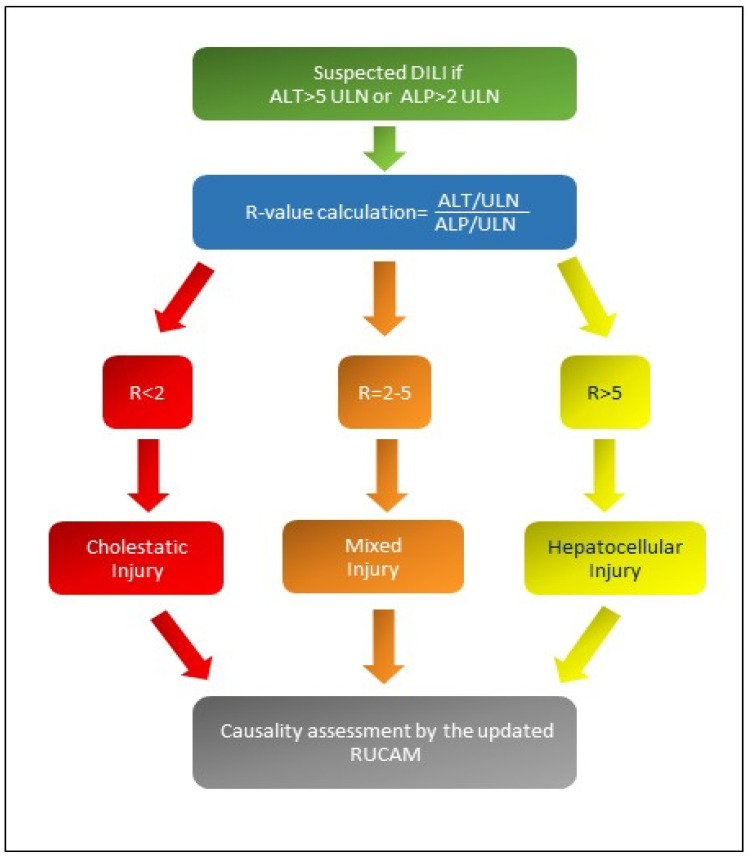
If R-value is greater than 5, a hepatocellular iDILI occurs; if R-value is less than 2, a cholestatic iDILI is diagnosed. Finally, if R-value is between 2 and 5, a mixed iDILI, cholestatic and hepatocellular, is present [5,6]. Abbreviations: ALT: alanine aminotransferase; ALP: alkaline phosphatase; ULN: upper limit of normal values; R: ratio; RUCAM: Roussel Uclaf Causality Assessment Method scale.

There are different patterns of iDILI according to R-value: Figure 1 shows the formula for R calculation and the different patterns of iDILI according to R-value [5,6]. At the same time, Table 1 explains the two assessments of iDILI causality for hepatocellular iDILI and mixed/cholestatic iDILI by an update of the RUCAM scale proposed by Danan and Teschke in 2015 [6]: if the resulting causality score was ≤ 0, iDILI was excluded; if 1–2, it was unlikely; if 3–5, it was possible; if 6–8, it was probable; finally, if it was ≥9, iDILI was highly likely.

DIC accounts for 20 to 40% of cases of iDILI, and after discontinuation of the involved drug, the resolution of liver disease is slow compared to hepatocellular injury [7,8].

The main histological pictures of DIC are cholestatic hepatitis, vanishing bile duct syndrome (VBDS), acute and chronic cholestasis, and finally immune checkpoint inhibitor-related cholangiopathies; indeed, immune-mediated large-ducts cholangitis, immune-mediated small-ducts cholangitis, and immune-mediated cholecystitis have been described among anti-programmed death-ligand 1/anti-programmed cell death protein 1 (anti-PD-L1-1/Anti-PD-1) immune-related hepatobiliary disorders [9,10,11]. 

Among the predisposing conditions for developing iDILI are some significant genetic factors. The human leukocyte antigen (HLA) class I and II genes are emerging issues in the predisposition to certain forms of iDILI since the HLA have a crucial role in the host immune response; for example, this association is described between HLA-DRB1*15 and co-amoxiclav and specific HLA variants and liver toxicity due to flucloxacillin [12].

Other most studied genetic factors predisposing to iDILI are defects of the biliary transporters of the adenosine triphosphate (ATP)-binding cassette (ABC) transporter family. 

The liver plays a key role in the systemic clearance of lipophilic drugs and their metabolites through their excretion in the bile. Many medications cause cholestasis through interaction with liver transporters. Interestingly, the etiology of DIC involves altered bile transporters and allergic immune mechanisms: examples include ductular forms of iDILI, such as VBDS, where a drug or its metabolite triggers an immune response against biliary cells [13]. 

The ATP-dependent canalicular transporters, members of the multidrug resistance (MDR) protein (MRP) family, primarily regulate the process of clearance of drugs and metabolites by the liver; they are the bile salt export pump (BSEP) protein (*ABCB11* gene), the multidrug resistance protein-2 (MRP2, *ABCC2* gene) capable of causing bile salt-independent flow by excretion of glutathione, the multidrug resistance-1 protein (MDR1, *ABCB1* gene) carrying organic cations, and the multidrug resistance-3 protein (MDR3, *ABCB4* gene) [14,15]. 

**Figure 2:**
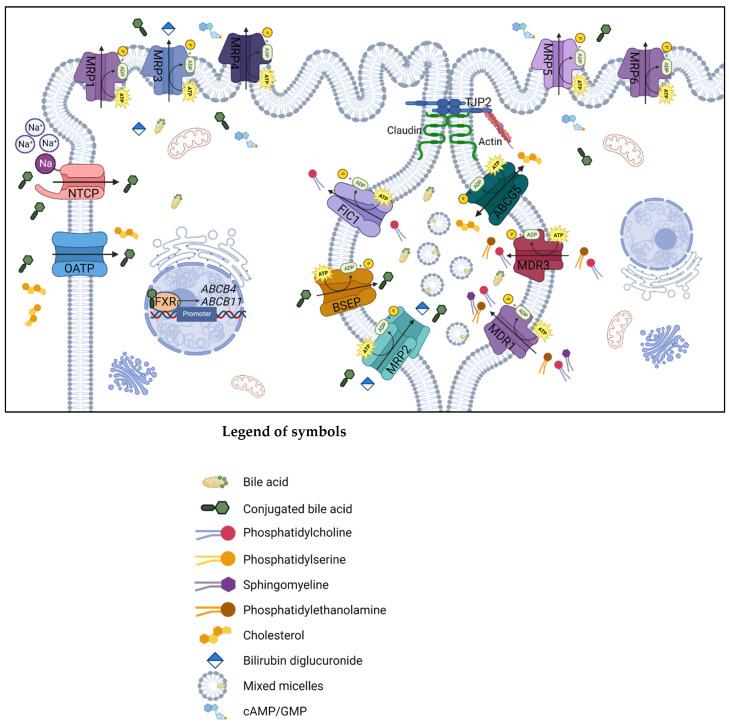
Simplified representation of the proteins expressed by the genes involved in the main pathway of synthesis, transport, and reuptake of BAs in the biliary canaliculus and hepatocyte membrane. Created with BioRender.com, accessed on 10 February 2023. The ATP-binding cassette superfamily proteins are localized in the biliary canaliculus, and mutations in these genes are associated with cholestatic liver diseases: the *ABCB1* gene encodes the transmembrane transporter P-glycoprotein MDR1 that works as a drug-transport pumping out a varied range of xenobiotics from cells, and it is required for PC secretion into bile. *ABCC2* synthesizes MRP2, an integral membrane glycoprotein expressed mainly in the canalicular membrane of liver cells. It transports endogenous and exogenous anionic conjugates from hepatocytes to bile. MRP2 is also involved in the resistance of cancer cells to chemotherapeutic drugs. The *ABCB11* gene is responsible for BSEP synthesis; this protein transports taurocholate and other cholate conjugates from hepatocytes to the bile, resulting in the primary determinant of bile formation and bile flow. Finally, the *ABCB4* gene-related protein, MDR3, is a lipid translocase or flippase causing the PC translocation to the inner leaflet of the membrane into the bile; in the absence of phospholipids such as PC, BAs cannot form mixed micelles, and the bile results are extremely hydrophobic, causing cell inflammation, bile ductular reaction and fibrosis. Abbreviations: ABCG5, ATP-binding cassette sub-family G member 5; BSEP, bile salt export pump; cAMP/GMP, cyclic adenosine monophosphate/cyclic guanosine monophosphate; FIC1, familial intrahepatic cholestasis type 1; FXR, farnesoid X receptor; MDR, multidrug resistance protein; MRP, multidrug resistance-associated protein; NTCP, sodium/taurocholate cotransporting polypeptide; OATP, organic anion-transporting polypeptide; TJP2, tight junction protein 2.

Figure 2 shows the simplified representation of the ABC transporter proteins involved in synthesizing and transferring bile acids (BAs) in the liver cells.

Mutations in the *ABCB11* and *ABCB4* genes are historically linked to progressive familial intrahepatic cholestasis (PFIC) type 2 and type 3. PFIC includes many autosomal recessive disorders of newborns and the young, characterized by intrahepatic cholestasis due to bile synthesis and transport defects. PFIC can evolve into portal hypertension, liver failure, and liver cancer within the first years of life [16]. Furthermore, single or compound heterozygosis mutations in the *ABCB11* and *ABCB4* genes are involved in different disease phenotypes, sometimes non-progressive [17,18,19]. These are benign recurrent intrahepatic cholestasis (BRIC), intrahepatic cholestasis of pregnancy (ICP), low-phospholipid-associated cholelithiasis (LPAC), and DIC [15,20]. Finally, mutations in PFIC genes increase the risk of the onset of hepatobiliary cancers [21].

Concerning *ABCB11*, a link between attenuated BSEP activities and DIC occurrence has been suggested. The inhibition of BSEP due to drug activity should lead to reduced secretion and retention of BAs within hepatocytes, resulting in cholestasis.

Instead, MDR3-protein, expressed by *ABCB4*, although not a drug transporter, helps the secretion of phosphatidylcholine in the bile; mutations in *ABCB4* result in the exposure of the biliary epithelium to the toxic detergent effects of BAs, predisposing to the risk of DIC [22].

Conversely, mutations in the *ABCC2* gene cause loss of MRP2, resulting in Dubin–Johnson syndrome, a clinical picture characterized by conjugated hyperbilirubinemia and pigment deposit in the liver. Defects in MRP2 synthesis induce a significant reduction in the bile flow, worsening cholestasis and increasing individual susceptibility to DIC by impairing the clearance of xenobiotics and drug metabolites from hepatocytes to the gut by BAs’ efflux [23].

Finally, the P-glycoprotein MDR1, expressed by the *ABCB1* gene, is involved in the transport of a broad spectrum of structurally variable substances; it not only confers multidrug resistance to cancer cells by actively excreting various chemotherapeutics, but also affects the pharmacokinetic properties of various hydrophobic therapeutic drugs, including alkaloids, flavonoids, and other hydrophobic natural toxic compounds [24].

## 2. Aims and Literature Search

Here, we reviewed the leading molecular pathways between the DIC and *ABCB1*, *ABCC2*, *ABCB11*, and *ABCB4* genes, the links with the other clinical forms of familial intrahepatic cholestasis, and, finally, the main cholestasis-inducing drugs.

The literature search, carried out by 31 December 2022, included e-Pub published articles (peer-reviewed original articles, reviews, and meta-analyses) with the following search terms:

“Benign Intrahepatic Cholestasis”, “Familial Intrahepatic Cholestasis”, “Drug-Induced Cholestasis”, “Drug-Induced Liver Injury”, “Progressive Familial Intrahepatic Cholestasis”, “ABCC2”, “ABCB1”, “ABCB11”, “ABCB4”, “Bile salts”, “Bile acids”, “BSEP”, “MDR3”, “MDR1”, “MRP2”, “PFIC”, “DIC”, “DILI”, “genetics of cholestasis”.

In addition to researching articles published via e-Pub, we consulted OMIM^®^ (Online Mendelian Inheritance in Man^®^), an international resource of human genes and genetic phenotypes (http://omim.org, accessed on 31 December 2022), for publications on hereditary cholestasis and DIC-DILI to ensure more excellent research coverage.

We also consulted PharmGKB, a National Institutes of Health (NIH)-funded resource that provides information about how human genetic variation affects medication response concerning the association between mutations in *ABCC2*, *ABCB1*, *ABCB11*, *ABCB4,* and drug phenotypes (https://www.pharmgkb.org/, accessed on 31 December 2022).

Moreover, we assessed the structures of canalicular efflux transport proteins through UniProt Protein Data Bank (PDB) while we visualized the proteins and residues by PyMOL (https://pymol.org/2/, accessed on 31 December 2022). As a result, residues are labeled as colored spheres according to their actual classification on Varsome (https://varsome.com/ accessed on 31 December 2022) [25,26].

Finally, according to the American College of Medical Genetics and Genomics (ACMG) standards [27], the variants in *ABCB1*, *ABCC2*, *ABCB11*, and *ABCB4* cited in the article were categorized into five categories: pathogenic (P); likely pathogenic (LP); variants of uncertain significance (VUS); likely benign (LB); and benign (B).

We recovered additional articles from references.

## 3. Molecular Bile Excretory and Metabolisms Pathways of Canalicular Efflux Transport Proteins in Drug-Induced Cholestasis

### 3.1. Multidrug Resistance Protein 1

The hepatobiliary transporters are expressed at the two polar surfaces of liver cells: the transporters located at the basolateral surface are responsible for the cellular uptake. In contrast, those located at the apical surface are responsible for the secretion of BAs and other bile products into bile. After canalicular secretion, bile composition undergoes further modification in the bile canaliculi, involving reabsorption and secretion processes maintained by the apical and basolateral transports system in cholangiocytes. The main actions of these transport systems are therefore to coordinate the bile formation and the biliary secretion of cholephilic products, including the hepatic clearance of drugs [14]. In addition to the uptake systems, several ATP-dependent efflux pumps are present in the basolateral hepatocyte membrane. These transporters belong to the family of multidrug resistance proteins (MRPs). The bile formation, the secretion of BAs, and hepatic drug clearance across the canalicular hepatocyte membrane are instead mediated by ABC transporters [28]. In normal liver, ABC transporters participate in different cellular processes, mediating the excretion of lipids, BAs, and organic anions, contributing to bile formation, and enabling the cells to keep the intracellular levels of toxic compounds low.

MDR1 is another ATP-dependent transporter, encoded by the *ABCB1* gene. MDR1 is a canalicular membrane transporter that works as a functional barrier and an efflux transporter in various tissues. The role of this molecule is in driving the excretion of multiple drugs, especially of quaternary cationic amines, converging them into the bile: xenobiotics, cytotoxins, cyclosporine, erythromycin, chlorpromazine, anticancer drugs, cardiac glycosides, and HIV protease inhibitors. Variants in this gene have been shown to alter the expression and/or function of MDR1 [29]. Overexpression of P-glycoprotein in tumor cells leads to multidrug resistance or enhances drug excretion, inhibiting the reaching of a pharmacologically active concentration, because of MDR1 regulation of pharmacokinetics of several anticancer drugs [30,31]. Moreover, *ABCB1* can induce iDILI by drug accumulation and toxicity in liver cells due to its decreased expression or competition for binding sites with other compounds [32,33,34]. 

### 3.2. Multidrug Resistance Protein 2

MRP2 is a molecule located on the canalicular membrane of hepatocytes and belongs to the ABC transporter family, which plays a key role in the biliary excretion of a wide variety of organic anions such as glutathione, bilirubin, leukotrienes, and sulfated or glucuronidated BAs [35]. 

The metabolism of BAs within hepatocytes leads to sulfated and glucuronidated BAs, particularly in cholestatic conditions [36,37]. In addition, BAs’ derivatives are driven into bile throughout the multidrug resistance protein MRP2, encoded by the *ABCC2* gene, or back into the sinusoids by MRP3 and MRP4, which are two different systems that help to reduce potentially cytotoxic intracellular BAs’ concentration in hepatocytes [38,39]. 

Mutations of the *ABCC2* gene cause Dubin–Johnson syndrome, characterized by hyperbilirubinemia, elevated BAs’ levels, increased coproporphyrin isomer I in the urinary excretion, and deposition of melanin-like pigment in liver cells, but normal liver function [40]. 

### 3.3. Bile Salt Export Pump

The protein encoded by the *ABCB11* gene is a member of the ABC transporter family. These transporters have two membrane-spanning domains (MSDs) that confer substrate specificity and two nucleotide-binding domains (NBDs) involved in ATP binding and hydrolysis. The *ABCB11* gene is localized on chromosome 2 (2q31) and it encodes canalicular BSEP, a protein of the hepatocytes membrane. Naturally, *ABCB11* expression is induced by BAs and is mediated by the farnesoid X receptor (FXR), which binds as a heterodimer with the retinoid X receptor (RXR). It eliminates unconjugated and conjugated BAs from hepatocytes into the bile against a concentration gradient and requires hydrolysis of ATP for transport activity. This function is essential for keeping the potentially cytotoxic BAs at a low intracellular level in hepatocytes [41,42,43,44,45]. 

Mutations in the *ABCB11* gene are responsible for several different genetic forms of cholestasis: PFIC2, formerly known as Byler’s syndrome, BRIC2, and other acquired forms of cholestasis. PFIC2 is a rare autosomal recessive disease affecting patients harboring a homozygous or a compound heterozygous status for *ABCB11* variations during early childhood. As a result, patients have less than 1% of primary BAs in their bile, decreased bile flow, accumulation of BAs inside the hepatocyte, hepatocellular damage, and an increased risk of hepatocellular carcinoma (HCC) [46,47].

Depending on the mutation, the BSEP protein may be completely absent from the canalicular membrane, present within the membrane but not functional, or trapped in the hepatic cytosol due to an abnormal conformation of the protein [45]. 

Inhibition of BSEP-mediated transport is one of the mechanisms that cause DIC [48,49]. In fact, drugs that inhibit export on the canalicular side can lead to cholestasis in susceptible people [14]. The drug classes primarily associated with DIC are anti-infectious, antidiabetic, anti-inflammatory, psychotropic and cardiovascular agents, steroids, and other miscellaneous drugs [50]. Patients with mutations in genes that encode BSEP or MDR3 have a 3-fold increased risk of cholestatic iDILI [51].

The molecular mechanisms of DIC have been investigated since the 1980s, but they still need to be clarified. One theory is that the mechanism for BSEP inhibition involves binding to the intracellular ATP-binding site or the substrate-binding site. Studies carried out in isolated membrane vesicles, hepatocyte cultures, and in bile duct cannulated murine models indicate that cholestatic drugs can inhibit bile secretion and bile acid transport at many levels, including uptake, defect of efflux transporters expressed on the surface of liver cells, whose FXR is a key regulator, and canalicular efflux [52,53,54].

### 3.4. Multidrug Resistance Protein 3

MDR3, or ATP-binding cassette (ABC) subfamily B member 4 (ABCB4), is a membrane-associated transport P-glycoprotein almost exclusively expressed in the liver. Its expression is restricted to the apical membrane of hepatocytes, even if mRNA levels were also found in other normal tissues [55]. 

This protein is encoded by the *ABCB4* gene, located on chromosome 7q21.1, and it consists of 27 coding exons with 2 cytoplasmic NBDs and 2 transmembrane domains, each with 6 transmembrane segments [56]. It works as a transporter and as an energy-dependent “floppase”, translocating phospholipids (PL) of the phosphatidylcholine (PC) family from the inner to the outer side of the lipid bilayer of the canalicular membrane of hepatocytes and is thereby responsible for PL secretion into the bile (Figure 2). It regulates the development of mixed micelles and accumulation of BAs resulting in bile production with deleterious detergent properties to the membranes of cholangiocytes and hepatocytes [57,58]. 

*ABCB4* gene mutations are responsible for several cholestatic diseases with a heterogeneous clinical spectrum. It has been demonstrated that *ABCB4* allelic status correlates with the phenotype and severity of liver disease [59]. 

According to the recent functional classification, variations in the *ABCB4* gene can be classified as nonsense mutations or class I; missense mutations affecting the maturation of protein or class II; missense mutations altering the activity of *ABCB4* protein or class III; missense mutations affecting the stability of the MDR3 protein or class IV; variations without identifiable effect on molecular function and expression or class V [60]. 

Approximately 300 *ABCB4* diseases causing variants have been reported, usually with homozygous status or compound-heterozygous with nonsense mutations in rapidly progressive cholestatic liver disease and complications, and with heterozygous status in less severe forms [59]. These are PFIC 3, LPAC, ICP, adult biliary fibrosis or cirrhosis, chronic cholangiopathy, iDILI, and transient neonatal cholestasis (TNC) [61]. Furthermore, recent genome-wide association studies (GWAS) have found an association between *ABCB4* variants and primary hepatobiliary malignancies [62,63,64]. 

Studies performed during these last years concerning the iDILI phenotype have shown that *ABCB4* deficiency predisposes to DIC: xenobiotics that inhibit these P-glycoproteins can also induce cholestasis in predisposed patients with *ABCB4* variants. Treatment with drugs that potentially inhibit the expression and/or function of *ABCB4*, such as sirolimus, cyclosporine, verapamil, or vinblastine, may deregulate biliary FC excretion in patients with a genetic *ABCB4* deficiency that is otherwise clinically silent. However, *ABCB4* transporter expression is altered in most cases of DIC, independent of *ABCB4* variants [65].

## 4. Canalicular Efflux Transport Proteins and Compounds Involved in Drug-Induced Cholestasis

### 4.1. Multidrug Resistance Protein 1

Members of the ABCC and ABCB subfamilies play a crucial role in the bile formation process. 

Five of the twelve transporters of the ABCC subfamily have been identified in liver tissue. The best characterized are the apical MRP2 (*ABCC2*) and its basolateral homologues MRP1 (*ABCC1*) other than MRP3 (*ABCC3*). 

Members of the ABCB subfamily that are expressed in the liver also include members of the family of MDR P-glycoproteins such as MDR1 (gene symbol *ABCB1*) [66].

Drugs interfering with these transporters can lead to an intracellular accumulation of bile/bile constituents, potentially harmful accumulation, and the development of cholestatic liver cell damage.

Table 2 shows the main features of *ABCB1*, *ABCC2*, *ABCB11*, and *ABCB4* biliary transporters, the respective main inhibitors drugs, the DIC-related mutations, and the disease variant predictions. 

The transporters’ system can also be altered by pre-existing hepatic disease/genetic factors which contribute to the developing of drug-induced cholestasis in susceptible individuals [14]. Both MDR1 and MRP2 are variably implicated in idiosyncratic iDILI [67]. 

MDR1 (*ABCB1*), expressed in the basolateral hepatocyte membrane, is a critical cell membrane protein responsible for clearing many foreign substances out of the cells [28]. The exact mechanism by which MDR1 contributes to hepatic bile formation remains to be established, but it is known to be implicated in the excretion of drugs into bile. Even the exact contribution of MDR1 to hepatic drug clearance has yet to be made available. MDR1 is present [28] in various tissues with an excretory function, such as small intestinal cells and renal cells of the proximal tubules, where it is a major determinant of drug disposition and toxicity [68]. Since MDR1 is involved in transporting many drugs with hepatotoxic potential, such as amiodarone, HIV protease inhibitors, and chemotherapeutics, it has been speculated that an altered hepatic MDR1 function might contribute to decreased biliary elimination of compounds, promoting hepatotoxicity [14]. Up to date, the primary known activity of MDR1 is its central role in conferring multidrug resistance to cancer cells by actively excreting structurally diverse chemotherapeutic compounds from cells; MDR1 is believed to be one of the critical molecules that cause multidrug resistance in cancer [30,31,69]. 

In addition, *ABCB1* has been suggested to play a role in specific hepatotoxicity forms because its reduced expression or competition for binding sites with other medicines may cause drug accumulation and toxicity [34]. 

However, contrasting data exist on the real role of these transports and their genetic alteration in developing iDILI. For example, Hung et al. [28] found no associations between genetic variants of MDR1 and MRP2 and the risk of iDILI, which is consistent with other previous studies [70,71]. Specifically, Bai et al. explored the possible association of ABCC gene polymorphisms with susceptibility to antituberculosis-related iDILI in 746 Western Han eligible patients with tuberculosis; they found a 15.8% rate of iDILI in the study population, while the *ABCC2* rs3740065 polymorphism, the sex, and the baseline level of alanine aminotransferase were the only independent risk factors for antituberculosis iDILI (*p* values of 0.008, 0.014, and <0.001, respectively) [71]. 

Concerning antiretroviral drugs, mutations in this transporter described in humans have been studied in patients with iDILI due to nevirapine. In African iDILI cases and a United States patient group, a significantly decreased frequency of the *ABCB1* single-nucleotide polymorphism (SNP) c.3435C>T (rs1045642) was reported. In contrast, a subsequent study on a separate group of European patients did not confirm this association [32,33,72]. However, in the last updated version of the human genome reference (GRCh38.p14) adopted by dbSNP, the wild-type nucleotide in this position is A, of which alternative alleles are G, C, and T; minor allele frequencies for the G allele are 0.7785 (African) and 0.4805 for European, which means it is the most frequent among the African population. The other two alleles are very rare [73].

Moreover, a study led by Saab et al. [67] demonstrated that MDR1 and MRP2 are variably implicated in idiosyncratic iDILI only in the presence of inflammatory reaction iDILI-induced. In the absence of an inflammatory stress, none of the drugs tested (Trovafloxacin, nimesulide, telithromycin, and nefazodone) in the study modulated the efflux activity of MRP2 and MDR1. Conversely, an in vitro inflammatory stimulus mediated by tumor necrosis factor-alpha and lipopolysaccharide, along with several idiosyncratic drugs, modulates the hepatic drug transporters’ expression and their activity, confirming the important role of co-existing inflammatory stimuli in promoting the development of iDILI. In the presence of inflammatory stress, the authors, using microvolume cytometry, showed telithromycin and nefazodone inhibited the efflux activity of MDR1, while trovafloxacin, nimesulide, and nefazodone attenuated the efflux activity of MRP2 [67]. In conclusion, the association between *ABCB1* polymorphisms and iDILI is yet to be established with certainty. 

**Figure 3:**
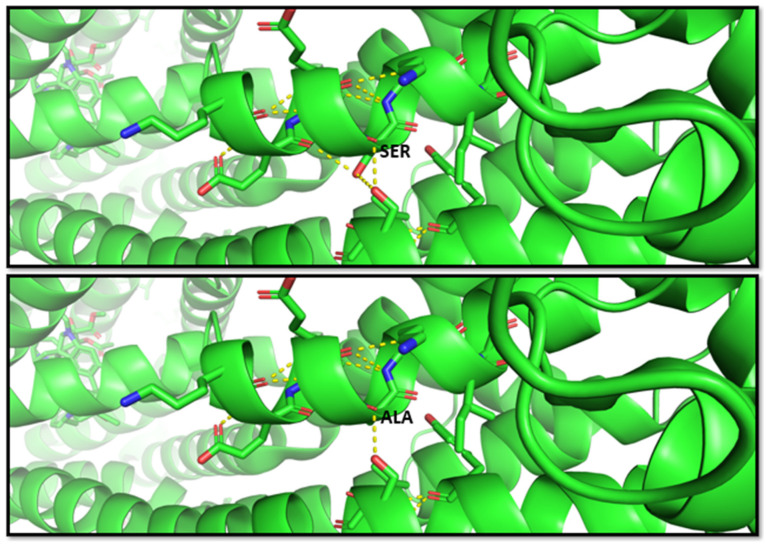
Molecular visualization of likely benign variant p.S893A in *ABCB1* (MDR1 protein) by using PyMOL, accessed on 30 January 2023. The residue change of amino acid serine to alanine causes two bond losses for –OH groups with the other two near residues in the helix. Transcript reference: ENST00000622132.5.

Another study by Fukunaga et al. [68], in 2016, indicated that the G allele of the SNP rs2032582 was significantly associated with atorvastatin-induced liver injury (odds ratio (OR) = 2.59)) and annotated in PharmGKB (ID 1448104351). The nucleotide substitution c.2677T > G causes an amino acid change p.S893A. Therefore, this variant is classified as benign from multiple sources such as Varsome; however, this residue change results in two bond losses, as shown in the molecular representation in Figure 3.

### 4.2. Multidrug Resistance Protein 2

MRP2 (*ABCC2*) is expressed in the canalicular part of hepatocytes and is responsible for the glucuronidated and sulfated BAs’ efflux. It is also implicated in transporting a broad spectrum of organic anions and drug substrates such as antibiotics and chemotherapeutic agents [66]. Several genetic variants may affect its activity, with some associated with Dubin–Johnson syndrome [74] or the susceptibility to develop diclofenac-induced hepatotoxicity, such as the *ABCC2* rs717620 polymorphism [75]. Daly et al. investigated the role of some polymorphisms in *UGT2B7*, *CYP2C8*, and *ABCC2* genes in 24 patients who had suffered diclofenac-related iDILI, compared with 48 intrahospital subjects taking diclofenac without developing a liver injury and 112 healthy controls. Within the investigated genotypes, the *ABCC2*variant c.-24C>T in 5′UTR was more common in patients with diclofenac-related iDILI compared with the hospital (odds ratio (OR) = 5.0, *p* = 0.005) and healthy controls (OR = 6.3, *p* = 0.0002). The authors concluded that allelic variants of *ABCC2* predispose to iDILI by forming and accumulating reactive metabolites [75]. A dysfunctional transporter gene is, in fact, in charge of both the individual’s susceptibility to developing drug liver injury as well as the severity of its presentation. In the study by Huang et al. [28], the *ABCC2* (MRP2) rs717620 T variant was associated with an increased risk of hyperbilirubinemia and mortality in patients with iDILI, confirming that ABC transporter genetic variations may play an essential role in iDILI. 

Fouassier et al. [76] proposed an additional mechanism of cholestatic cell damage. While they investigated the effect of bosentan on MRP2-mediated canalicular bile formation, they showed that bosentan stimulates and significantly increases MRP2-dependent bilirubin excretion but deeply inhibits BAs’ secretion. By this, the BAs secreted in the bile canaliculi are diluted below the concentration required for solubilization. The consequent physicochemical disequilibrium in bile composition may therefore be responsible for the decreased biliary phospholipid secretion even without canalicular genetic defects [76].

Recent studies have reported that rifampicin-induced hepatotoxicity is closely related to endoplasmic reticulum (ER) stress and MRP2 [77,78]. In addition, ER stress, caused by the accumulation of unfolded or misfolded proteins in ER, has been implicated in the pathogenesis of iDILI and rifampicin-induced liver injury [78,79]. 

Another study revealed two peculiar mechanisms that may contribute to hepatotoxicity caused by valproate, an antiepileptic drug used to treat bipolar disorder and anxiety. Valproate inhibits apical trafficking of MRP2, causing increased intracellular accumulation of MRP2 and decreased canalicular localization of MRP2. Moreover, valproate reduces zonula occludens-2 (ZO2) protein levels, causing tight junction disruption and hepatocyte depolarization. This aspect suggests that individuals with a pathological or acquired condition that impacts one of these pathways may exhibit increased susceptibility to iDILI. Valproate hepatotoxicity has been associated with mitochondrial toxicity, especially with inhibition of b-oxidation, which is unlikely to be the cause of impaired MRP2 trafficking and, consequently, accumulation of toxic MRP2 substrates [80]. 

**Figure 4:**
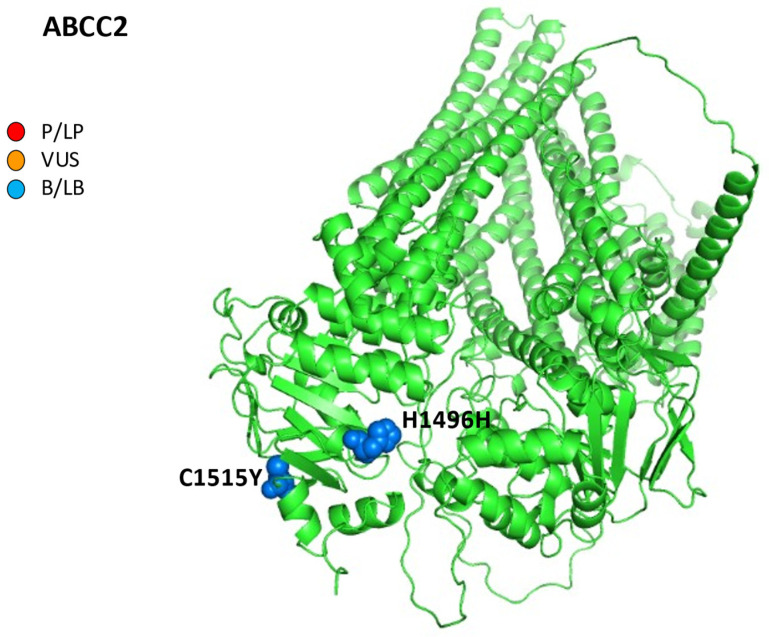
AlphaFold structure prediction of ATP-binding cassette sub-family C member 2. All five reported variations for *ABCC2* were mapped on available 3D structures, as assessed through UniProt. AF-Q92887-F1-model_v4 of ABCC2 protein in helix, turn, and beta strand conformation. Visualization of the protein and residues has been performed by PyMOL (https://pymol.org/2/, accessed on 30 January 2023). Here, residues are shown in the text: p.H1496H (c.4488C>T) and p.C1515Y (c.4544G>A) (ABCC2 NM_000392.5). Residues are labeled as colored spheres according to their actual classification on Varsome. P/LP, red spheres: pathogenic/likely pathogenic; VUS, orange spheres: variant at uncertain significance; B/LB, blue spheres: benign/likely benign.

Finally, as regards the reports of iDILI from chemotherapeutic agents, an association between *ABCC2* mutations and trabectedin has been reported: the authors described a case of a 60-year-old male patient affected by a pleiomorphic sarcoma on the right tibial crest. After two cycles of drugs, an irreversible mixed iDILI (cytolytic and cholestatic) occurred. Furthermore, the genes investigations revealed a deficient variant genotype in *ABCC2* (c.-24TT, c.4488CT, and c.4544GA), suggesting their causative role in the excretion of toxic metabolites of trabectedin (Figure 4) [81]. 

### 4.3. Bile Salt Export Pump

iDILI, especially DIC, are part of the less aggressive clinical manifestations of ABCB11 disease, mainly when variants result in heterozygous mutations [51].

In predisposing *ABCB11* genetic conditions, many drugs can induce downregulation of BSEP on the canalicular side, favoring the development of DIC. To understand the relationship between BSEP inhibition and iDILI, the inhibition of BSEP-mediated transport of taurocholic acid (TCA) by drugs in a vesicular BSEP inhibition assay has been examined in previous studies. Although false positives range from 7 to 38%, cyclosporine, rifampin, bosentan, troglitazone, sulindac, erythromycin, glibenclamide, and isoniazid/rifampicin directly, and estradiol and progesterone metabolites indirectly, inhibit BSEP activity in vitro by another ATP-dependent transporter, the MRP2 [54,82,83]. 

On the other hand, in vivo, not only causative mutations in *ABCB11* can cause DIC but also some polymorphisms. The most famous and studied is a valine-to-alanine exchange at the highly conserved position 444 (p.V444A) associated with a reduced expression of BSEP in hepatocytes: beta-lactam antibiotics, oral contraceptives, psychotropic drugs, and proton pump inhibitors can cause iDILI in carriers of p.V444A mutation [14,84].

**Figure 5:**
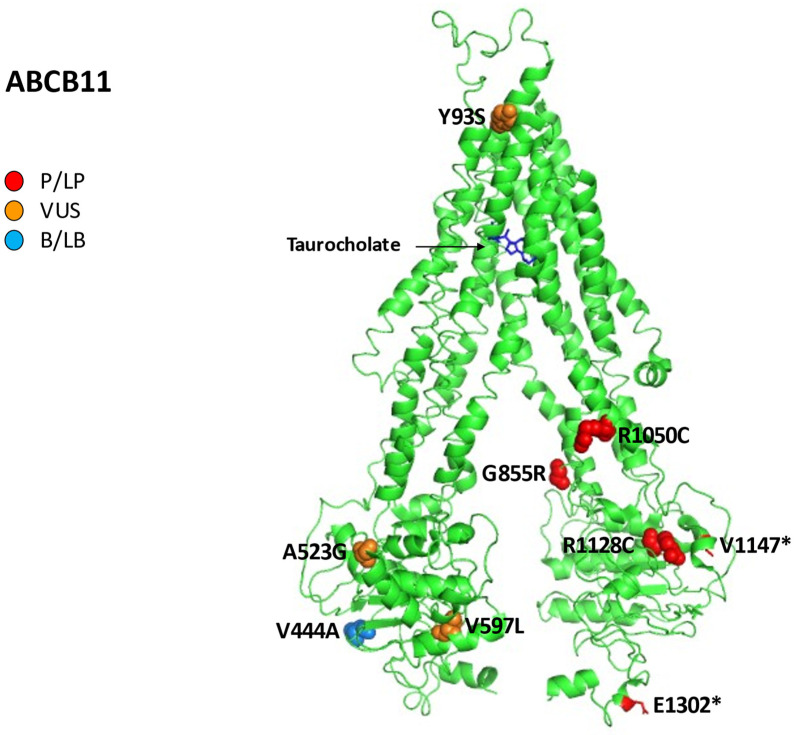
Human bile salt exporter ABCB11 in complex with taurocholate. All 12 reported variations for *ABCB11* were mapped on available 3D structures, as assessed through UniProt. Protein Data Bank (PDB) code 7e1a of BSEP protein in helix, turn, and beta strand conformation. Visualization of the protein and residues has been performed by PyMOL (https://pymol.org/2/, accessed on 30 January 2023). Residues are labeled as colored spheres according to their actual classification on Varsome [24].

Figure 5 reports all variations for ABCB11 linked to iDILI, mapping them on available 3D structures by UniProt and their Varsome classification.

In can be pointed out that the polymorphism p.V444A often increases the susceptibility for ICP and contraceptive-induced cholestasis in some female patients [84].

However, in a Japanese cohort of patients, p.V444A polymorphism in ABCB11 was not associated with iDILI, while the C allele in this variant increased the risk of liver injury by drugs containing a carbocyclic system with aromatic rings [85,86]. A Spanish study based on the national iDILI Registry of 188 cases (51 DIC, 48 mixed liver injury, 89 drug-induced hepatocellular injury) analyzed the presence of p.V444A, resulting in a higher frequency in patients compared to healthy controls (OR 1.6, 1.1–2.4, *p* = 0.01). However, homozygous p.V444A was significantly associated with iDILI (*p* = 0.01) only when causative drugs caused <50% BSEP inhibition [86].

p.V444A could not cause iDILI alone; the need for an additional factor to alter BSEP function resulting in liver injury is possible.

One of the medication categories most involved in developing iDILI and DIC is antituberculosis drugs. In a cohort of 89 Chinese patients with iDILI due to antituberculosis drugs matched with 356 iDILI-free subjects, there were no significant differences in p.V444A frequency between the two groups. However, in additional subgroup analysis, this polymorphism was linked with the DIC/iDILI-DIC mixed pattern of liver injury (OR = 3.84, 95% confidence interval (CI): 1.16–12.75, *p = *0.028, and OR = 2.51, 95% CI: 1.12–5.62, *p = *0.025, respectively) [87].

In another study, 23 patients with DIC and 13 with iDILI were tested concerning *ABCB11* ed *ABCB4* variants compared to 95 healthy controls. The results showed a correlation between two nonsynonymous p.D676Y and p.G855R mutations in *ABCB11* and DIC (drug-induced cholestasis). Furthermore, the polymorphism p.V444A was significantly more frequent in patients with DIC compared to iDILI subjects and the controls group (76 vs. 50 and 59%, respectively; *p *< 0.05). Here, the heterozygous p.D676Y in *ABCB11* was linked to the assumption of fluvastatin with hepatocellular cholestasis. Finally, the same study correlated two *ABCB4* p.I764L and p.L1082Q variants with DIC and iDILI, respectively [51]. 

Other drugs (e.g., glimepiride, pioglitazone, simvastatin), although potent BSEP inhibitors, are associated with a low risk of iDILI, suggesting other additional patient-related mechanisms of liver injury such as mitochondrial dysfunction as well as a diabetes condition [88]. 

Instead, the endothelin receptor antagonist bosentan, used in pulmonary hypertension, caused reversible, dose-dependent iDILI during clinical trials, while plasma BAs’ levels increased with the dose of the drug [89].

Moreover, some chemotherapy drugs, such as taxol, may be involved in drug-induced cholestasis by BSEP dysfunction [90].

The troglitazone, nefazodone, and benzbromarone drugs were removed from the market since they caused iDILI by mitochondrial toxicity and the inhibition of BSEP protein [90].

Drugs or herbal products can trigger a cholestasis attack in a predisposing genetic condition [91].

Fotoulaki et al. described the case of a 27-month-old girl who presented with jaundice initially attributed to iDILI. In the previous days, she had received cefprozil and a homoeopathic preparation of cantharidin for cystitis. The genetic test revealed a compound heterozygote for the missense mutation c.3148C>T (p.R1050C) previously associated with BRIC and for the nonsense mutation c.3904G>T (p.E1302*) previously associated with PFIC. The infant evolved in 5 years in end-stage liver disease needing liver transplantation [92].

Finally, mutations in the *ABCB11* gene can often cause different phenotypes of cholestasis in the same patient: in addition to DIC, ICP, PFIC, BRIC, and LPAC may be present. In relation to this, we described a case of a 20-year-old, homozygous for a missense variant in *ABCB11* (p.A523G), with persistent jaundice and DIC related to the oral contraceptive pill, who had evidence of advanced fibrosis on liver biopsy, compatible with a PFIC–BRIC mixed phenotype [93].

DIC had been associated not only with oral contraceptives but also with a contraceptive vaginal ring. In a patient with a prior history of ICP, the heterozygous pathogenic mutation c.3439_3440delGTinsTA (p.V1147*) and the heterozygous variant at uncertain significance (VUS) in *ABCB4* c.1846G>A (p.E616K) were detected; the patient resolved itching and jaundice one month after the vaginal ring removal [94].

In another cohort of 48 patients with cryptogenic cholestasis, we showed a DIC history in 35% of cases, itching in 27%, neonatal jaundice in 21%, juvenile cholelithiasis in 17%, personal or family history of ICP in 13%; interestingly, pathogenic/likely pathogenic mutations were found in 10 (21%) probands for 13 mutations: 6 in *ABCB11*, 2 in *ATP8B1*, 2 in *ABCB4*, and 3 in *TJP2*. The five pathogenic and likely pathogenic variants linked to DIC in the ABCB11 gene were:

- The three causative mutations c.278A>C (p.Y93S), c.1789G>C (p.V597L), and c.3382C>T (p.R1128C) combined in a female patient (29 years) with a history of DIC, ICP, juvenile cholelithiasis, and itching;
- The one homozygous mutation c.1568C>G (p.A523G) in two sisters (7 and 20 years), together with a history of neonatal jaundice, itching, and DIC (amoxicillin/clavulanic and contraceptives, respectively) [19].

Moreover, we found the polymorphism p.V444A in 40 cases (83.3% of the population studied) and an allele frequency (AF) significantly more frequent in our cohort of patients without pathogenic/likely pathogenic mutations in comparison with European and worldwide populations (81 vs. 60%: *p* = 0.008; 81 vs. 57%: *p* = 0.002), underlining its possible role in inducing cholestasis [19].

### 4.4. MDR3

Mutations in the *ABCB4* gene, encoding the MDR3 protein, have been linked to different phenotypes of ABCB4 deficiency, from PFIC3 to LPAC, ICP, hepatobiliary cancers, and iDILI [22].

MDR3 plays a vital role in the biliary secretion of phosphatidylcholine. Therefore, if MDR3 cannot translocate phospholipids across the canalicular membrane, the detergent effects of bile acids are toxic for cholangiopathies.

There are a lot of drugs able to reduce MDR3 activity in vitro. Aleo et al. demonstrated that more than 40% of 125 tested drugs could inhibit phosphatidylcholine efflux by MDR3 mediation in isolated human hepatocytes. Another studied category is antifungal drugs inhibiting MDR3 activity in vitro in transfected LLC-PK1 cells [95,96].

The inhibitors drugs of ABCB4 biliary transporter are reported in Table 1, including itraconazole, chlorpromazine, imipramine, haloperidol, ketoconazole, clotrimazole, and troglitazone [97].

Other treatments potentially able to inhibit the function or expression of MDR3 in vitro are sirolimus, cyclosporine, verapamil, and vinblastine [22]. 

In vivo, the main clinical pictures associated with the impaired expression of MDR3 are cholangitis cholestasis and the VBDS [90].

Subjects with mutations in the *ABCB11* e *ABCB4* gene have a 3-fold increased risk of DIC; instead, Lang et al. [51] linked the two non-synonymous *ABCB4* p.I764L and p.L1082Q variants with DIC and iDILI in a clinical study on 23 patients with DIC and 13 with IDILI, especially when patients assumed psychotropic drugs, such as risperidone and proton pump inhibitors, or selected antibiotics (the most frequent were antibacterials with the b-lactam ring) and oral contraceptives, by worsening of biliary phosphatidylcholine excretion. Regarding contraceptive-induced cholestasis, as mentioned above, it is often reported in women with a prior history of ICP (the goal of the pharmacological treatment of ICP with ursodeoxycholic acid is to improve maternal symptoms and reduce fetal adverse events) and is resolved by withdrawing the suspected drug [98,99].

**Figure 6:**
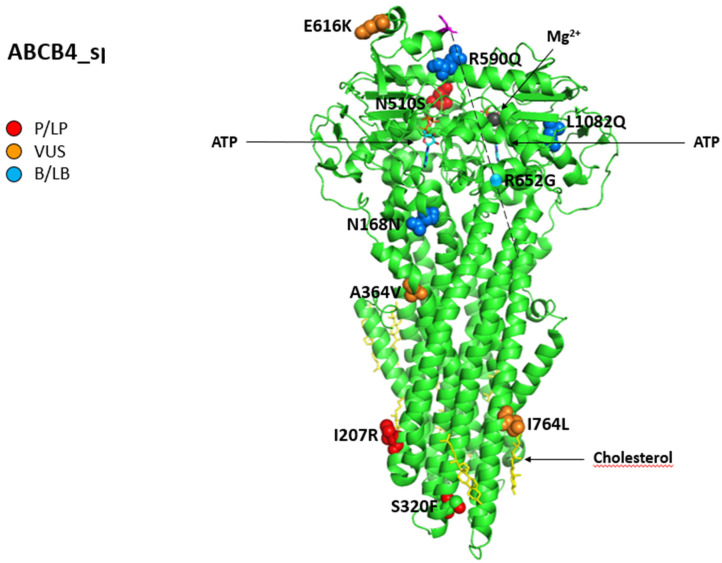
Structure of phosphatidylcholine translocator ABCB4. Cryo-EM structure of nanodisc-reconstituted human ABCB4 trapped in an ATP-bound state at a resolution of 3.2 Å. The nucleotide-binding domains form a closed conformation containing two bound ATP molecules, but only one of the ATPase sites has bound Mg^2+^ (dark grey). The transmembrane domains adopt a collapsed conformation at the level of the lipid bilayer. All 12 reported variations for *ABCB4* were mapped on available 3D structures, as assessed through UniProt. PDB code 6s7p of MDR3 protein in helix, turn, and beta-strand conformation. The spatial position of mutation R652G (single sphere) is inferred because no 3D structure was available for residues from 629 to 692 (magenta). The protein is in a lipid environment with a cholesterol structure in this representation. Visualization of the protein and residues has been performed by PyMOL (https://pymol.org/2/, accessed on 30 January 2023). Residues are labeled as colored spheres according to their actual classification on Varsome [26].

Figure 6 shows all variations for *ABCB4* linked to iDILI, mapping them on available 3D structures by UniProt and their Varsome classification.

Falcão et al. analyzed a cohort of twenty patients with suspected ABCB4 disease, with three of them presenting an iDILI.

The first case had a history of juvenile cholelithiasis since she underwent cholecystectomy at 17, and ICP was diagnosed twice. She also experienced a DIC due to the use of sodium valproate prescribed for bipolar disorder. Liver histology showed a VBDS with ductopenia, portal fibrosis, and foci of mild necroinflammatory activity. She resulted as a carrier of a compound heterozygosis in two missense *ABCB4* mutations, c.959C>T (p. S320F) and c.1529A>G (p. N510S). 

The second and third cases developed DIC after the assumption of antihypertensive drugs (atenolol and losartan) and rosuvastatin, respectively, with total normalization of the liver enzyme after they stopped the drugs. The genetic analysis revealed the presence of c.504C>T (p.N168N) in a homozygous state. In this cohort of patients, the authors found this mutation in two other cases, one with a history of altered GGT and the other with a history of cholecystectomy at 29 years due to cholelithiasis [100]. 

Instead, we found two pathogenic/likely pathogenic mutations in the coding region of *ABCB4* in two patients with a DIC history in the Bologna cohort of 48 cryptogenic cholestasis cases: the c.2014A>T (p.K672*) variant, previously undescribed, found in a 39-year-old woman and the c.1091C>T (p.A364V) variant, in a 57-year-old cirrhotic patient who also developed HCC [19].

A case of DIC during ciprofloxacin therapy was described in a subject with LPAC, carrier of *ABCB4*-related c.1954A>G (p.R652G), a homozygous missense mutation, already described in PFIC3 and ICP, combined to p.V444A in the *ABCB11* gene in a heterozygous state [101].

In the context of mutations of the *ABCB4* gene, not only different clinical phenotypes associated with DIC are reported, but the histology can also range. For example, in a case series of seven patients undergoing a liver biopsy, the authors detect a history of DIC in three cases with different clinical presentations:

- A 36-year-old woman with a background of Marfan syndrome and multiple sclerosis with cholelithiasis worsened cholestatic enzymes during dimethyl fumarate, which improved after drug discontinuation. Liver histology revealed ductopenia and minimal fibrosis without portal inflammation; a genetic test showed the pathogenic *ABCB4* variants c.620T>G, p.I207R), which are likely pathogenic in the heterozygous state; a variant c.1228G>C coexisted in the *CYP7A1* gene, having a pivotal role in cholesterol metabolism.
- A 46-year-old man with a history of multiple endoscopic retrograde cholangiopancreatography for recurrent cholelithiasis presented jaundice after co-amoxiclav intravenous administration; a liver biopsy suggested acute cholestasis on chronic ductopenia with moderate fibrosis and incomplete septa, while the two *ABCB4* variants, the intronic VUS 3507 + 10dupA and the pathogenic c.1769G>A, p.R590Q), were in the heterozygous state.
- A 45-year-old woman with progressive pruritus and a history of ICP, recurrent choledocholithiasis after a cholecystectomy performed at 22 years, received Rifampicin to control her itching; her liver function tests then worsened during the treatment and improved after suspension. Next-generation sequencing revealed the *ABCB4* variants: c.959C>T, p.S320F previously reported, likely pathogenic and c.2301dupT, p.T768Yfs*26 previously predicted, likely pathogenic combined to c.3149T>A p.I1050K)—VUS in *ATP8B1*, responsible for PFIC1. At histology, mild fibrosis, ductular reaction, ductopenia, and cholestatic rosettes were found [102].

However, unlike what happens for mutations in *ABCB11*, the inhibition of MDR3 in biliary phospholipid excretion may represent a risk factor for drug-induced cholestasis, although BA secretion is not altered. In clinical practice, mutations in *ABCB11* e *ABCB4* coexist, having a synergic effect in the induction of DIC [97].

Finally, the clinical cases reported above confirm the need to perform a genetic analysis to confirm or exclude mutations in *ABCB4* in the presence of DIC and personal or family history of PFIC, BRIC, LPAC, or ICP, such as contraceptive-induced cholestasis with increased GGT. 

## 5. Discussion

In recent years, the development and acquisition of new knowledge on the molecular structure and metabolism of drugs, on the one hand, and the functions and localization of the proteins present in hepatocytes and cholangiocytes, on the other, has made it possible to identify the basis of many cases of DIC previously thought to be cryptogenic cholestasis and to reduce the clinical consequences of iDILI.

This aspect has also proved helpful during drug development clinical trials.

EASL guidelines [91] consider inhibition of BSEP function with a daily dose of >100 mg, whatever the drug, a predominant liver metabolism by cytochrome P450 enzymes, the formation of reactive metabolites, and mitochondrial inhibition, as the main risk factors for iDILI. During the early phases of drug discovery and development, the EMA and FDA guidelines recommend the use by pharmaceutical companies of interaction testing of BSEP and multidrug resistance proteins such as MRP2 to assess the liver safety of molecular compounds.

Indeed, in cases of elevated transaminases or cholestasis enzymes during a trial, testing for inhibition of BSEP in vitro by the drug helps design the study’s safety plan and the metabolism of the drug; the relationship of the peak concentration at the end of infusion of the drug to the inhibitory affinity to BSEP is a crucial parameter to evaluate a potential iDILI. In addition, if BSEP interaction has been found during development, serum BAs’ levels investigation is an additional parameter for identifying iDILI. 

If drug metabolites are considerable, a vesicular BSEP assay should be supplemented with a system capable of drug metabolization, such as sandwiched cultured human hepatocytes.

As drug metabolites are substrates of MRP2, this canalicular export protein is also a risk factor for DIC; variants of MRP2 have been associated with iDILI [91].

Mutations in *ABCB4* can also induce cholestasis since molecular studies from tissue culture suggest that xenobiotics can inhibit P-glycoproteins [103].

*ABCB1* (MDR1) and *ABCB4* (MDR3) are two close genes probably developed by duplication of an ancestor gene. Still, they codify two canalicular proteins with distinct transport roles: MDR1 is a multispecific transporter of organic cations, while MDR3 is a phospholipids transporter [104].

Interestingly, *MDR1* and *MRP1* genes’ overexpression may be directly connected with tumorogenesis in HCC and resistance to apoptosis and some chemotherapies, especially doxorubicin, while a reduced expression, such as in liver cirrhosis and cholestatic diseases, could be prone to DIC. The outcomes in treating liver cancer observed during the natural history of liver cirrhosis and familial intrahepatic cholestasis are remarkable due to the risk of iDILI and resistance to antineoplastic drugs [105]. 

Canalicular ABC transporter expression is profoundly disturbed in most cases of cholestatic iDILI; certain drugs can induce DIC by a synergic perturbation of more canalicular ABC transporters’ expression, and liver histology often reveals a marked aberration of MRP2, MDR3, and BSEP transporter staining, especially in cholestatic iDILI due to antibiotics [65]. 

Thus, a deep understanding of the chemical characteristics of the drugs is required to reduce the risk of iDILI in drug development and commercialization, particularly lipophilicity and drug biotransformation and the patient’s clinical history with particular regard to the presence of risk factors such as LPAC, ICP, previous DIC, and FIC.

Indeed, the reactive metabolites of drugs can induce, in addition to oxidative stress, activation of signal transduction pathways and mitochondrial stress, and interference with bile acid transport, increasing the risk of necrosis or apoptosis in the liver of subjects with mutations in the ABC transporters MDR1, MDR3, MRP1, and BSEP [91]. 

On the other hand, to facilitate the activities of clinicians in reducing the risk and detecting iDILI, the National Institutes of Health and the US National Institute of Diabetes and Digestive and Kidney Diseases have developed a comprehensive database, constantly updated, available online (LiverTox http://livertox.nlm.nih.gov, accessed 10 February 2023), which provides complete information on diagnosis, cause, frequency, and molecular patterns of liver injury attributable to drugs and herbal and dietary supplements. 

Livertox is an extraordinary resource that provides a structured overview of key drug classes and for selecting alternative treatments that replace a suspected compound that has induced iDILI in a patient, reporting all published cases in the literature for each drug or herb [106].

In addition, PharmGKB, an NIH-funded database, provides information about how human genetic variation affects medication response concerning the association between mutations in ABCC2, ABCB1, ABCB11, ABCB4, and drug phenotypes.

## 6. Conclusions

Genetic-based DIC due to mutations in ABCB11, ABCB4, ABCC2, and ABCB1 represents a diagnostic challenge for the community of hepatologists, and its detection requires a high index of suspicion.

The differential diagnosis is with suppurative and non-suppurative cholangiopathies, such as primary biliary cholangitis, sarcoidosis, autoimmune hepatitis, primary sclerosing cholangitis, secondary sclerosing cholangitis, IgG4-associated cholangitis, and finally other cholangiopathies such as malignant cholangiopathy, lymphoma, systemic mastocytosis, neutrophilic cholangitis, eosinophilic cholangitis, and Langerhans cell histiocytosis [107].

Furthermore, it should be underlined that variants in the genes responsible for PFIC are not only in differential diagnosis with autoimmune liver diseases, but also represent a cofactor of the progression of liver damage. Kruk et al., in fact, described the role of variant ABCB4 c.711A>T in the modulation of liver injury in 456 patients with PBC. Patients carrying this variant more frequently had cirrhosis, increased serum transaminases, GGT, and ALP; patients without cirrhosis at baseline were at a high risk of developing advanced fibrosis during the follow-up [108].

Investigating the presence of different clinical phenotypes of intrahepatic cholestasis in the patient’s personal or family history, such as PFIC, BRIC, ICP, juvenile cholelithiasis, and LPAC, helps the diagnosis of DIC due to defects in bile canalicular transporters.

These conditions are not only typical of early childhood but can also occur in adolescence or adulthood. 

Finally, knowledge of the molecular features of the supposed drug and its metabolites can guide the diagnosis of DIC, especially if the risk conditions listed above are present in the patient’s history.

## Figures and Tables

**Table 1:** An updated RUCAM scale for the liver injury and cholestatic/mixed liver injury of iDILI/HILI, adapted with permission from Danan and Teschke [6].

Type of DILI				
Items	Hepatocellular	Score	Mixed/Cholestatic	Score
(1) Time to onset from the beginning of the drug/herb	5–90 days (rechallenge: 1–15 days) 90 days (rechallenge: >15 days)	+2 +1	5–90 days (rechallenge: 1–90 days) 90 days (rechallenge: >90 days)	+2 +1
Alternative: Time to onset from cessation of the drug/herb	≤ 15 days (except for slowly metabolized chemicals: >15 days)	+1	(except for slowly metabolized chemicals: ≤30 days)	+1
(2) Course of liver exams after cessation of the drug/herb	ALT decrease ≥ 50% within 8 days ALT decrease ≥ 50% within 30 days No information or continued drug use ALT decrease ≥ 50% after the 30th day ALT decrease < 50% after the 30th day or recurrent increase	+3 +2 0 0 −2	ALP decrease ≥ 50% within 180 days ALP decrease < 50% within 180 days No information, persistence, increase, or continued drug/herb use	+2 +1 0
(3) Risk factors	Alcohol use, current drinks/d: >2 for women, > 3 for men Alcohol use, current drinks/d: ≤2 for women, ≤ 3 for men Pregnancy Age ≥ 55 years Age < 55 years	+1 0 +1 +1 0	Alcohol use, current drinks/d: >2 for women, > 3 for men Alcohol use, current drinks/d: ≤2 for women, ≤ 3 for men Pregnancy Age ≥ 55 years Age < 55 years	+1 0 +1 +1 0
(4) Concomitant drug(s)/herb(s)	None or no information	0	None or no information	0
	Concomitant drug/herb with incompatible time to onset	0	Concomitant drug/herb with incompatible time to onset	0
	Concomitant drug/herb with compatible or suggestive time to onset	−1	Concomitant drug/herb with compatible or suggestive time to onset	−1
	Concomitant drug/herb known as hepatotoxin and with compatible or suggestive time to onset delete marking right side above	−2	Concomitant drug/herb known as hepatotoxin and with compatible or suggestive time to onset delete marking right side above	−2
	Concomitant drug/herb with evidence for its role in this case (positive rechallenge or validated test)	−3	Concomitant drug/herb with evidence for its role in this case (positive rechallenge or validated test)	−3
(5) Search for alternative causes	Group I (7 causes) HAV: Anti-HAV-IgM HBV: HBsAg, anti-HBc-IgM, HBV-DNA HCV: Anti-HCV, HCV-RNA HEV: Anti-HEV-IgM, anti-HEV-IgG, HEV-RNA Hepatobiliary sonography/colour Doppler sonography of liver vessels/endosonography/CT/MRC Alcoholism (AST/ALT ≥ 2) Acute recent hypotension history Group II (5 causes) Complications of underlying disease(s) such as sepsis, metastatic malignancy, autoimmune hepatitis, chronic hepatitis B or C, PBC or PSC, genetic liver diseases Infection suggested by PCR and titer change for: CMV EBV HSV VZV		Group I (7 causes) HAV: Anti-HAV-IgM HBV: HBsAg, anti-HBc-IgM, HBV-DNA HCV: Anti-HCV, HCV-RNA HEV: Anti-HEV-IgM, anti-HEV-IgG, HEV-RNA Hepatobiliary sonography/colour Doppler sonography of liver vessels/endosonography/CT/MRC Alcoholism (AST/ALT ≥ 2) Acute recent hypotension history Group II (5 causes) Complications of underlying disease(s) such as sepsis, metastatic malignancy, autoimmune hepatitis, chronic hepatitis B or C, PBC or PSC, genetic liver diseases Infection suggested by PCR and titer change for: CMV EBV HSV VZV	
	Evaluation of groups I and II All causes-groups I and II—reasonably ruled out The 7 causes of group I ruled out 6 or 5 causes of group I ruled out Less than 5 causes of group I ruled out Alternative cause highly probabl	+2 +1 0 −2 −3	Evaluation of groups I and II All causes-groups I and II—reasonably ruled out The 7 causes of group I ruled out 6 or 5 causes of group I ruled out Less than 5 causes of group I ruled out Alternative cause highly probable	+2 +1 0 −2 −3
(6) Previous hepatotoxicity of the drug/herb	Reaction labelled in the product characteristics Reaction published but unlabelled Reaction unknown	+2 +1 0	Reaction labelled in the product characteristics Reaction published but unlabelled Reaction unknown	+2 +1 0
(7) Response to unintentional reexposure	Doubling of ALT with the drug/herb alone, provided ALT below 5 ULN before reexposure Doubling of ALT with the drug(s)/herb(s) already given at the time of first reaction Increase of ALT but less than ULN in the same conditions as for the first administration Other situations	+3 +1 −2 0	Doubling of ALP with the drug/herb alone, provided ALP below 2 ULN before reexposure Doubling of ALP with the drug(s)/herb(s) already given at the time of first reaction Increase of ALP but less than ULN in the same conditions as for the first administration Other situations	+3 +1 −2 0

Results: >8 points, definitive; 6–8 points, probable; 3–5 points, possible; 1–2 points, unlikely; <0 points, excluded. Abbreviations: RUCAM, Roussel Uclaf Causality Assessment Method; iDILI, idiosyncratic Drug-Induced Liver Injury; HILI, Herb-Induced Liver Injury; ALT, alanine aminotransferase; AST, aspartate aminotransferase; ALP, alkaline phosphatase; HAV, hepatitis A virus; HCV, hepatitis C virus; HBc, Hepatitis B core; HBsAg, Hepatitis B antigen; HBV, hepatitis B virus; HEV, hepatitis E virus; CMV, cytomegalovirus; EBV, Epstein Barr virus; HSV, herpes simplex virus; VZV, varicella zoster virus; CT, computerized tomography; MRC, magnetic resonance cholangiography; ULN: upper limit of normal values; PBC, primary biliary cholangitis; PSC, primary sclerosing cholangitis.

**Table 2:** Genes, proteins, cell position, function, inhibitors drugs, mutations, disease variants’ prediction, and transcripts of ABCB1, ABCC2, ABCB11, and ABCB4 biliary transporters, according to the ACMG standards [26].

Genes, Protein, Cell Position	Function	In Vitro Inhibitors of ABC Transporters and In Vivo Liver Injury-Related Drugs	Variants, Prediction, Transcript
ABCB1 MDR1 Canalicular membrane	It works both as a functional barrier and as an efflux transporter. In addition, it drives the excretion of multiple drugs, especially of quaternary cationic amines, converging them into bile	Cyclosporine, Erythromycin, Chlorpromazine, Anticancer drugs, HIV protease inhibitors, Amiodarone, Antituberculosis Drugs, Nefazodone, Telithromycin, Carvedilol, Clarithromycin, Itraconazole, Lapatinib, Verapamil	c.2677T>G p.S893A (LB) c.3435T>G p.I1145M (VUS) **Transcript: NM_000927.4**
ABCC2 MRP2 Canalicular membrane	ATP-dependent efflux of numerous drugs, organic anions, such as glutathione, bilirubin, leukotrienes, and sulfated or glucuronidated BAs	Anticancer drugs (Trabectedin) Benzbromarone, Bosentan, Cyclosporine, Diclofenac, Efavirenz, Probenecid, Rifampicin, Valproate	c.-24C>T in 5’UTR **^#^** c.4146+154A>G **^#^** c.4488C>T p.H1496H (B) c.1249G>A p.V417I (B) c.3972C>T p.I1324 (B) c.4544G>A p.C1515Y (B) **Transcript: NM_000392.5**
ABCB11 BSEP Canalicular membrane	It eliminates unconjugated and conjugated BAs from hepatocytes into the bile against a concentration gradient. This function is essential for keeping the potentially cytotoxic BAs at a low intracellular level in hepatocytes	Benzbromarone, Bosentan, Cyclosporine, Isoniazid, Rifampicin, Glitazones, Statins, Sulindac, Erythromycin, Glibenclamide, Nefazodone, Progesterone metabolites, Estradiol 17b-Glucuronide, Taxol, Cefprozil combined with homoeopathic preparation of cantharidin, Antibacterials with b-lactam ring	c.1331T>C p.V444A (B) c.2026G>T p.D676Y **^§^** (LB) c.278A>C p.Y93S (VUS) c.1568C>G p.A523G (VUS) c.1789G>C p.V597L (VUS) c.2563G>A p.G855R (LP) c.3449_3440delGTinsTA p.V1147* (LP) c.3148C>T p.R1050C (P) c.3382C>T p.R1128C (P) c.343 c.3904G>T p.E1302* (P) **Transcript: NM_003742.4**
ABCB4 MDR3 Canalicular membrane	It works as “floppase”, translocating PL of the PC family from the inner to the outer side of the lipid bilayer of the canalicular membrane of hepatocytes; it is responsible for PL secretion into the bile, regulating the development of mixed micelles	Itraconazole, Chlorpromazine, Imipramine, Haloperidol, Ketoconazole, Clotrimazole, Troglitazone, Sirolimus, Cyclosporine, Verapamil, Vinblastine, Atenolol, Losartan Risperidone, Proton pump inhibitors, Antibacterials with b-lactam ring, Oral contraceptives, Ciprofloxacin	c.504C>T p.N168N (B) c.1769G>A p.R590Q (B) c.1954A>G p.R652G (B) c.3245T>A p.L1082Q (LB) c.1091C>T p.A364V (VUS) c.1529A>G p.N510S (VUS) c.1846G>A p.E616K (VUS) c.2290A>C p.I764L (VUS) c.620T>G p.I207R (LP) c.2014A>T p.K672* (LP) c.959C>T p.S320F (P) c.2301dupT p.T768Yfs*26 ^§^ (P) **Transcript: NM_000443.4**

Abbreviations: P: pathogenic; LP: likely pathogenic; VUS: variant at uncertain significance; LB: likely benign; B: benign. **^#^** no protein-coding variant. **^§^** not shown in PyMOL. Data obtained from the references in this article.

## Data Availability

Not applicable.

## Abbreviations

ABC	ATP-binding cassette	MSDs	membrane-spanning domains
ABCG5	ATP-binding cassette sub-family G member 5	NBDs	nucleotide-binding domains
ACMG	American College of Medical Genetics and Genomics	LPAC	low-phospholipid-associated cholelithiasis
AF	allele frequency	MDR	multidrug resistance protein
ALF	acute liver failure	MRP	multidrug resistance protein
ALT	alanine aminotransferase	NGS	next-generation sequencing
ALP	alkaline phosphatase		
Anti-PD-1	anti-programmed cell death protein 1	NIH	National Institutes of Health
Anti-PDL-1	anti-programmed death-ligand 1	NTCP	sodium taurocholate cotransporting polypeptide
AP	amino phospholipids	OATP	organic anion-transporting polypeptide
ASBT	apical sodium-dependent bile acid transporter	OMIM	online mendelian inheritance in man
ATP	adenosine triphosphate	OR	odds ratio
B/LB	benign/likely benign	P/LP	pathogenic/likely pathogenic
BAs	bile acids	PBC	primary biliary cholangitis
BSEP	bile salt export pump	PSC	primary sclerosing cholangitis
BRIC	benign recurrent intrahepatic cholestasis	PDB	protein data bank
CCA	cholangiocarcinoma	PE	phosphatidylethanolamine
CI	confidence interval	PFIC	progressive familial intrahepatic cholestasis
DIC	drug-induced cholestasis	PMID	PubMed-Indexed for MEDLINE
iDILI	idiosyncratic drug-induced liver injury	PS	phosphatidylserine
ER	endoplasmic reticulum	RXR	retinoid X receptor
FIC 1	familial intrahepatic cholestasis deficiency type 1 protein	RUCAM	Roussel Uclaf Causality Assessment Method scale
FXR	farnesoid X receptor	ULN	upper limit of normal
GWAS	genome-wide association studies	SNP	single-nucleotide polymorphism
HBCs	hepatobiliary cancers	TJP2	tight junction protein 2 gene
HCC	hepatocellular carcinoma	TNC	transient neonatal cholestasis
HILI	herb-induced liver injury	VBDS	vanishing bile duct syndrome
HLA	human leukocyte antigen	VUS	variant at uncertain significance
ICP	intrahepatic cholestasis of pregnancy	ZO2	zonula occludens-2
LT	liver transplantation		
